# Atypical atrial myxomas in two asymptomatic patients: a case report

**DOI:** 10.1186/1476-7120-7-45

**Published:** 2009-09-08

**Authors:** Nicholas A Charokopos, Efthymia Rouska, Christodoulos Pliakos, Efstathios D Pagourelias, Panagiotis Artemiou, Christoforos Foroulis, Nikolaos Papadopoulos

**Affiliations:** 1 Department of Thoracic and Cardiovascular Surgery, AHEPA University Hospital, Medical School, Aristotle University of Thessaloniki, Thessaloniki, Greece; 2 First Cardiology Department, AHEPA University Hospital, Medical School, Aristotle University of Thessaloniki, Thessaloniki, Greece

## Abstract

**Background:**

Atypical cardiac myxomas are a rare occurrence and may present with a variety of clinical manifestations depending on the morphology and location.

**Case presentation:**

Two cases of cardiac myxomas atypically located in asymptomatic patients, diagnosed by transthoracic and transoesophageal echocardiography, are presented. In the first case a myxoma is located under the anterior mitral valve leaflet and in the second case a myxoma is located in the right atrium.

**Conclusion:**

We emphasize the leading role of transthoracic and transoesophageal echocardiography in the diagnosis of intracavitary cardiac tumours.

## Background

Myxoma is the most common primary cardiac neoplasm and accounts for approximately one-half of all primary cardiac tumours. A left atrial myxoma was first described in a post-mortem examination in 1845. The specific signs and symptoms produced by a cardiac myxoma depend on its anatomic location. Approximately 75% of these tumours arise from the left atrium and 18% from the right atrium. The few remaining tumours originate from atypical sites such as left or right ventricle and valves [1-3].

We report two cases of atypical cardiac myxomas (originating from mitral valve and the right atrium), which were diagnosed by means of transthoracic (TTE) and transoesophageal echocardiography (TOE).

## Case presentation

### Case 1

A 30- year-old man was admitted to our hospital with a history of hypertension. He had no history of cardiac symptoms, syncope or fever, and his past medical history was unremarkable.

**Figure 1:**
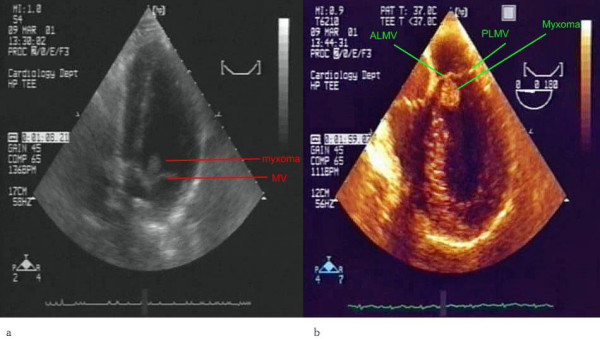
**Myxoma originating from the anterior mitral valve leaflet**. **a**. Transthoracic echocardiographic study, apical 2 chamber view. Myxoma seen attached at the ventricular surface of the anterior mitral valve leaflet (dimensions 1,99 × 1,1 cm). **b**. Transoesophageal echocardiographic study, 4 chamber view. Left ventricular myxoma arizing from a thin stalk attached at the ventricular surface of the anterior mitral valve leaflet. ALMV refers to anterior leaflet of mitral valve and PLMV refers to posterior leaflet.

On admission, patient's heart rate was regular and his blood pressure was high, 170/100 mmHg. Blood biochemistry was revealed to be normal. The 12-lead electrocardiogram demonstrated regular sinus rhythm and the chest x-ray was normal. As a part of the cardiologic workup a transthoracic echocardiography (TTE) examination was performed. This revealed a mobile mass, 1.1 × 1.99 cm, attached to the ventricular surface of the anterior mitral leaflet (Figure 1a). It was a mass with multiple lobules. Evidence of inflow obstruction of left ventricular outflow tract (LVOT) was present. There was no mitral regurgitation and the left atrial size was normal. On transoesophageal echocardiography (TOE) this mobile LV mass was measured 1.7 × 2.0 cm, appeared peduncular and was attached to the anterior leaflet of the mitral valve (Figure 1b). The mass had multiple lobes, was highly echogenic and appeared to obstruct LVOT.

The appearance of the LV mass was consistent with a myxoma arising from an atypical location. The patient was referred for surgical excision of the mass. Pathologic examination confirmed the diagnosis of myxoma. At 6-month follow-up, the patient was asymptomatic, and a TTE was within normal limits.

### Case 2

**Figure 2:**
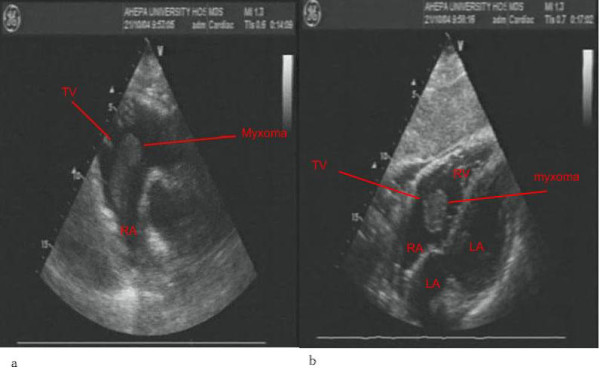
**Myxoma in the right atrium**. **a**. Transthoracic echocardiographic study, parasternal short axis view. Right atrial (RA) myxoma seen protruding across the tricuspid valve (TV) into the right ventricle. **b**. Transthoracic echocardiographic study, subcostal 4 chamber view. Right atrial (RA) myxoma seen protruding across the tricuspid valve (TV) into the right ventricle (RV) during diastolic period. LA refers to left atrium and LV to left ventricle.

A 65-year-old woman was admitted to our hospital in order to have surgery for a bronchocele. She complained for dyspnoea worsening on exertion. Her past medical history was unremarkable illustrating no signs of heart or respiratory disease. On examination, the heart sounds and blood pressure were normal. A 12-lead electrocardiogram and chest x-ray appeared to be normal. As a part of her endocrinological workup a TTE study was performed detecting a mobile mass of 6.5 × 5.5 × 4.5 cm in the right atrium (RA). The mass was highly echogenic, attached to the atrial wall by a narrow pedicle and appeared protruding through the tricuspid valve into the right ventricle (Figure 2). The interatrial septum was normal in appearance. The presence of a large RA mass was also confirmed by TOE. It was consistent with a myxoma arising from an atypical location in the RA. No other intracardiac masses were noted.

The mass was removed surgically and pathologic examination confirmed that it was a myxoma. A follow-up transthoracic echocardiogram was within normal limits. The patient was doing well 10 months after surgery.

## Conclusion

Clinically, intracavitary tumours present with a dyad of clinical signs comprising obstruction and embolization [4]. Dominating symptoms and signs relate to tumour location, which seems to be the main factor predicting obstructive sequelae and influencing embolic sites. Based on this concept, left atrial myxomas commonly cause mitral valve obstruction mimicking symptoms of rheumatic heart disease [5], while right-sided myxomas, being extremely rare, may present with nonspecific signs and symptoms, including right heart failure secondary to RV outflow tract obstruction [6]. Although various clinical signs and symptoms produced by cardiac myxomas have been reported in the literature, asymptomatic giant cardiac myxomas of the right atrium, such as the one described in our second patient are very rare [7].

Echocardiography has made a profound impact on the diagnosis and management of cardiac myxomas. With M-mode and two-dimensional echocardiography the preoperative diagnosis of this pathology has increased to 90% [2]. Even though TTE studies usually yield adequate images for the diagnosis of cardiac tumors, the use of TOE may offer valuable assistance in patients with poor acoustic windows by virtue of confirming tumor attachment site and dimensions, detecting other masses, and defining any obstruction to flow [2,8]. With TOE we particularly evaluated the posterior left atrial wall, which often is not well displayed on TTE examination [4]. Especially in case of left atrial myxomas associated with mitral stenosis, TOE can fairly easy discriminate an atrial thrombus from a myxoma.

Although echocardiography is the modality of choice in screening for cardiac masses, magnetic resonance imaging (MRI) and electron-beam computed tomography (EBCT) provide additional information about tissue characteristics and allow an excellent overview of the cardiac and paracardiac morphology. The advantage of these techniques is that they provide sectional views of cardiac and thoracic structures without superposition in any plane [1,9-11].

About 75% of myxomas originate in the left atrium (LA) while 15-20% of them are located in the RA. The usual site of attachment is the area of the fossa ovalis in the LA [3]. However, myxomas can be found in atypical locations, arising from the posterior or anterior LA wall or the atrial appendage [9]. Rarely myxomas originate from the ventricle (3-4%) arising from the mitral valve. Atypical locations and multiple myxomas occur most frequently in cases of familial myxomatosis [10]. Mitral valve myxomas may be localized to the anterior mitral leaflet, posterior mitral leaflet or mitral annulus. Usually the tumour is localized to the atrial-lower pressure side of the mitral valve [3,12-14].

The treatment of choice for myxomas is surgical removal. Operative mortality ranges from 0% to 3% in multiple series. Recurrence rates are between 1% and 3% for sporadic myxomas, as a result of incomplete resection, versus 12% for familial myxomas [6].

In conclusion echocardiography is of primary importance, among the non-invasive diagnostic tools. It has facilitated the ante-mortem diagnosis of cardiac myxomas even if the patient is asymptomatic and the myxoma arises from an atypical location, as it was observed in our two patients.

## Consent

Written informed consent was obtained at first visit from all patients for publication of this case report and all accompanying images. A copy of the written consent is available for review by the Editor-in-Chief of this journal.

## Competing interests

The authors declare that they have no competing interests.

## Authors' contributions

NAC and ER conceived the case report, collected the data, reviewed literature and wrote the manuscript. CP and EDP revised the article for important intellectual content and edited the final version. CP and ER performed the ultrasounds and participated in the analysis and interpretation of data. NAC, CF, AP and NP performed the surgical dissection of myxomas. All authors read and approved the final manuscript.
